# cAMP competitively inhibits periplasmic phosphatases to coordinate nutritional growth with competence of *Haemophilus influenzae*

**DOI:** 10.1016/j.jbc.2023.105404

**Published:** 2023-10-29

**Authors:** Kristina Kronborg, Yong Everett Zhang

**Affiliations:** Department of Biology, University of Copenhagen, Copenhagen, Denmark

**Keywords:** cAMP, natural competence, *Haemophilus influenzae*, periplasmic acid phosphatase, nucleotide, nucleoside, NAD, NMN, NadN, hel, e(P4), AphA

## Abstract

Most naturally competent bacteria tightly regulate the window of the competent state to maximize their ecological fitness under specific conditions. Development of competence by *Haemophilus influenzae* strain Rd KW20 is stimulated by cAMP and inhibited by purine nucleotides, respectively. In contrast, cAMP inhibits cell growth, but nucleotides are important for KW20 growth. However, the mechanisms underlying the abovementioned reciprocal effects are unclear. Here, we first identified a periplasmic acid phosphatase AphA_Ec_ of *Escherichia coli* as a new cAMP-binding protein. We show cAMP competitively inhibits the phosphatase activities of AphA_Ec_ and its homolog protein AphA_Hi_ in the KW20 strain. Furthermore, we found cAMP inhibits two other periplasmic nonspecific phosphatases, NadN_Hi_ (which provides the essential growth factor V, NAD) and Hel_Hi_ (eP4, which converts NADP to NAD) in KW20. We demonstrate cAMP inhibits cell growth rate, especially *via* NadN_Hi_. On the other hand, the inhibitory effect of purine nucleotide AMP on competence was abolished in the triple deletion mutant Δ*hel*_*Hi*_Δ*nadN*_*Hi*_Δ*aphA*_*Hi*_, but not in the single, double deletion or complemented strains. Adenosine, however, still inhibited the competence of the triple deletion mutant, demonstrating the crucial role of the three phosphatases in converting nucleotides to nucleosides and thus inhibiting KW20 competence. Finally, cAMP restored the competence inhibited by GMP in a dose-dependent manner, but not competence inhibited by guanosine. Altogether, we uncovered these three periplasmic phosphatases as the key players underlying the antagonistic effects of cAMP and purine nucleotides on both cell growth and competence development of *H. influenzae*.

In the phenomenon of natural competence, a bacterium initiates a genetic program to take up external DNA and integrate it into its chromosome. It is currently believed that natural competence is critical for horizontal gene transfer, and thus bacterial genome evolution and the emergence of multidrug resistant bacteria (1). Understanding when and how bacteria become competent is therefore essential to mitigate the detrimental effect of multidrug resistant bacteria in health care. Most of the naturally competent bacteria have a tightly regulated time window to become competent in response to environmental stresses, the regulatory mechanisms of which are intensively studied (2). Here, we studied the molecular interplay of the stimulatory and inhibitory effects of 3′,5′-cAMP and nucleotides, respectively, on the competence development in the model organism *Haemophilus influenzae* Rd KW20 (hereafter KW20) (3, 4).

The competence program of KW20 begins with the production of 3′,5′-cAMP upon bacterial perception of stresses, such as exhaustion of carbon source at the end of the growth phase in rich medium (5). In the laboratory, KW20 competence is often induced by shifting log-phase cells grown in rich brain heart infusion medium supplemented with hemin and NAD (sBHI) medium to M-IV minimal medium that lacks carbon sources and consequently induces cAMP production (6). Additionally, high concentrations (1–10 mM) of exogenously added cAMP to growing cells induce growth arrest and competence development of KW20 (7). cAMP binds to the carbon catabolite protein or cAMP receptor protein (CRP_Hi_), to stimulate the production of the master competence regulator Sxy_Hi_ (8, 9). Furthermore, the mRNA of *sxy*_*Hi*_ encodes a long noncoding 5′ sequence that is currently believed to perceive some intracellular signals (*e.g.,* purine nucleotides, see below) to control the translation of *sxy*_*Hi*_ (9). Consistently, genetic changes in the *sxy*_*Hi*_ mRNA leader region produce constitutively competent KW20 mutants (5, 9). The ternary complex of cAMP-CRP_Hi_-Sxy_Hi_ stimulates the expression of 25 genes that are involved in taking up external DNA and thus competence development in KW20 (10). Thus, cAMP and CRP_Hi_ link the competence development of KW20 to the quality of environmental nutrients. However, it remains unclear why KW20 cell growth is arrested in a rich medium when a high concentration of extracellular cAMP is applied.

By contrast to carbon starvation, the purine nucleotides AMP and GMP inhibit the competence development of KW20 (4). Both purine nucleo*t*ides and nucleo*s*ides inhibit competence development when they are added early during the M-IV medium induced competence program (3). Previous studies suggest a model wherein external purine nucleosides enter the cytosol of KW20 and participate in the purine nucleotide biosynthesis pathway. This metabolic change is perceived by factors including PurR, or the Sxy_Hi_ mRNA 5′-end structure to repress the translation of Sxy_Hi_ *via* a still mysterious mechanism that may involve a riboswitch (3). Nucleotides added in a later stage of M-IV induced competence program failed to inhibit competence and the mentioned constitutively active Sxy_Hi_ mutants are not inhibited by nucleotides (3), indicating that nucleotides affect Sxy_Hi_ production. However, the connections between cAMP, nucleotide metabolism, and competence development are incompletely understood.

Besides competence, nucleotides (purine and pyrimidine) are abundant in the native niche of KW20, mucus (11). Nucleotides are energetically expensive to synthesize, and KW20 encodes an incomplete *de novo* synthesis pathway for pyrimidine nucleotides, thus requiring external pyrimidines to grow.

Furthermore, KW20 cannot synthesize NAD, an essential molecule for all organisms. Therefore, NAD (the so-called growth factor V for KW20), nicotinamide mononucleotide (NMN), or nicotinamide riboside (NR) is required to support the growth of KW20. A periplasmic phosphatase NadN_Hi_ is essential for the conversion of NAD to NMN and subsequently to NR (12), which then traverses into the cytosol *via* PnuC (12, 13). Lastly, the oxygen carrier hemin (essential growth factor X) is required for KW20 to grow. The utilization of hemin was thought to require an outer membrane lipoprotein Hel_Hi_ or e(P4). However, it was later found that Hel_Hi_ converts NADP to NAD, and potentially further to NMN and NR (14, 15). Importantly, both NadN_Hi_ and Hel_Hi_ show nonspecific phosphatase activities toward the nucleotides (14, 15). Therefore, nucleotides affect not only competence development but also cell growth in KW20.

In this study, we started with a systematic screening of cAMP-binding proteins in *Escherichia coli* K12 and, surprisingly, identified the periplasmic acid phosphatase AphA_Ec_. We show that cAMP competitively inhibits the AphA_Ec_ catalytic activity. Furthermore, we find that cAMP competitively inhibits the homolog protein AphA_Hi_ in KW20, and in addition, also Hel_Hi_, and NadN_Hi_, consistent with the inhibitory effect of cAMP on KW20 growth rate. A combined deletion of the three genes *aphA*_*Hi*_*, nadN*_*Hi*_*, hel*_*Hi*_ made KW20 immune to the inhibition of competence development by nucleotides, but not by nucleosides, suggesting that all three proteins together control competence development *via* cleaving nucleotides to nucleosides. Finally, we find that cAMP *via* its inhibition of the three phosphatases restores the competence inhibited by nucleotides, but not by nucleosides. Altogether, we reveal an intricate interplay of cAMP, nucleotides, and the three phosphatases in coupling cell growth with competence development in KW20 and potentially other related organisms.

## Results

### A proteome-wide screening identified the periplasmic acid phosphatase AphA as a novel cAMP binding protein in *E. coli*

**Figure 1:**
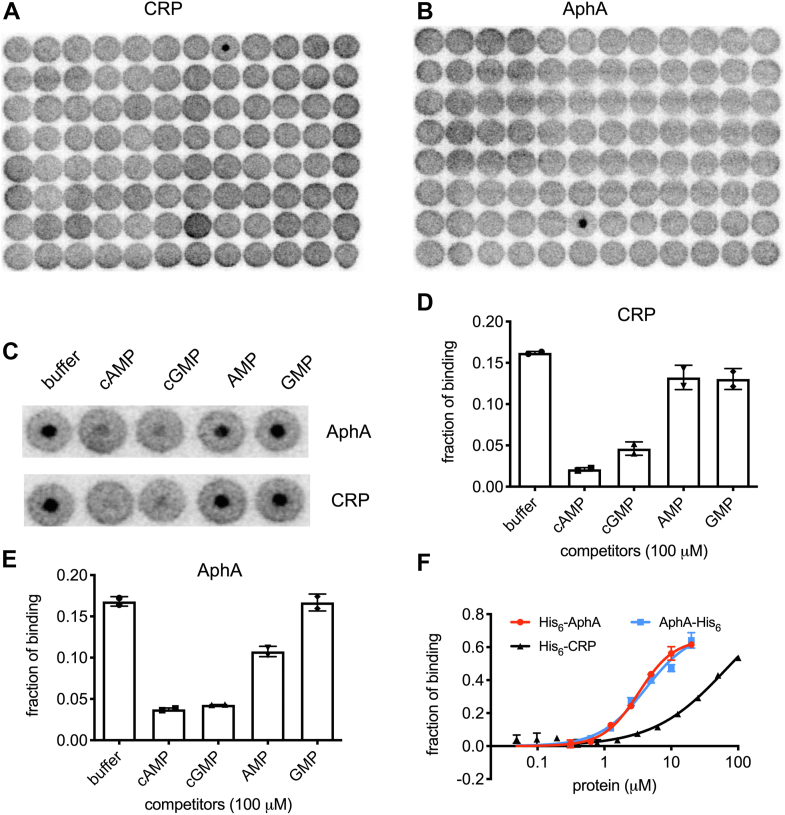
**cAMP binds to AphA of *Escherichia coli*.***A* and *B*, autoradiography of the two DRaCALA screening plates that identified CRP_Ec_ and AphA_Ec_ as cAMP-binding proteins. *C*, DRaCALA-based competition assay by using whole-cell lysates harboring overproduced AphA_Ec_ or CRP_Ec_, with the presence of buffer, or cold cAMP, cGMP, AMP, and GMP (each at 100 μM). *D* and *E*, quantitation of the p^32^-α-cAMP binding fractions from panel (*C*). Two biological replicates were performed. The average of binding fraction and the SD are shown. *F*, determination of the K_d_ values of cAMP binding to His_6_-AphA, AphA-His_6_, and His_6_-CRP proteins, by using DRaCALA (17, 18). Two biological replicates were performed, and the average of binding fraction and SD are plotted.CRP, cAMP receptor protein.

**Figure S1:**
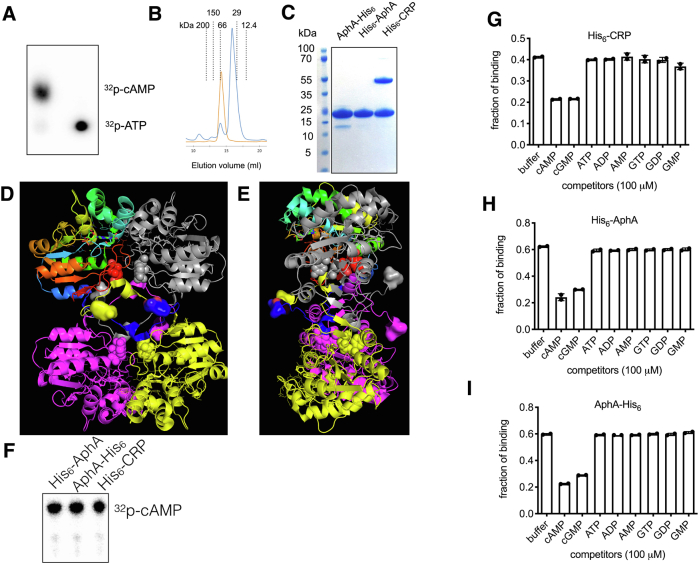
*A*, TLC plate showing the synthesis of ^32^p-labeled cAMP [p^32^-α-cAMP] from p^32^-α-ATP. *B*, the FPLC profiles of N- (*orange*) and C- (*blue*) 6-histidine–tagged AphA_Ec_. The sizes of the standard proteins are marked on the *top*. *C*, an SDS-PAGE gel picture of purified His_6_-AphA_Ec_, AphA_Ec_-His_6_, and His_6_-CRP_Ec_ proteins. *D*, structure of *Escherichia coli* AphA_Ec_ (2B82) presented in cartoon model with the N and C termini shown in *surface and sphere models*, respectively. The four monomers were colored differently. *E*, side view of the structure of AphA_Ec_ (2B82). *F*, TLC plate showing that the p^32^-α-cAMP was not degraded by AphA_Ec_ (10 μM) after 20 min at 37 ^o^C. *G*-*I,* quantitation of DRaCALA competition assay by using purified His_6_-AphA_Ec_, AphA_Ec_-His_6_, and His_6_-CRP_Ec_, with the presence of buffer, cold cAMP, cGMP, ATP, ADP, AMP, and GTP, GDP, GMP (each at 100 μM). Two biological replicates were performed, and the average and SD are shown.

The well-studied second messenger cAMP is critical for bacterial carbon metabolism, pathogenesis, and virulence (16). Presently, the only known target protein of cAMP in *E. coli* is CRP_Ec_ (or CAP). To explore if cAMP has additional effector proteins, we performed a proteome-wide screening of cAMP-binding proteins using DRaCALA (17, 18). First, cAMP was synthesized from p^32^-α-ATP by using the truncated recombinant *E. coli* CyaA protein (19) (conversion ratio >85%, Fig. S1*A*). The ordered ASKA strain collection (20) was used to overexpress proteins of *E. coli* K-12 MG1655, and whole-cell lysates were prepared as before (21). Radioactively labeled p^32^-α-cAMP was then mixed with individual whole lysates to screen for cAMP-binding proteins. CRP_Ec_ gave a strong binding signal (Fig. 1*A*), validating the screening method. Besides CRP_Ec_, AphA_Ec_, a nonspecific periplasmic phosphatase, also gave a strong binding signal (Fig. 1*B*; see other DRaCALA screening plates in Supplementary document S1).

To confirm the binding, the two ASKA strains overproducing CRP_Ec_ and AphA_Ec_ were used in a DRaCALA-based competitive binding assay. Various cold nucleotides (100 μM) were added to the binding reactions. Figure 1, *C*–*E* shows that cold cAMP, and also cGMP, effectively outcompeted the binding of p^32^-α-cAMP to both CRP_Ec_ and AphA_Ec_. On the other hand, the known AphA_Ec_ substrates AMP and GMP were less effective (see below), indicating that the binding of cAMP to CRP_Ec_ and AphA_Ec_ was specific.

### cAMP binds to AphA_Ec_ with a low-micromolar affinity

AphA_Ec_ is a periplasmic protein with an N-terminus (Nt) signal peptide. The ASKA plasmid produces a recombinant AphA_Ec_ with a six-histidine (His_6_) tag N terminal to the signal peptide and a C-terminal (Ct) GLCGR peptide (20). To understand how cAMP binds to AphA_Ec_, we first removed the signal peptide and fused a His_6_ tag at either the Ct- or Nt of AphA_Ec_ (AphA_Ec_-His_6_ and His_6_-AphA_Ec_, respectively). Both proteins were purified to homogeneity *via* tandem affinity purification and size-exclusion chromatography (SEC). The SEC profiles (Fig. S1*B*) suggested that AphA_Ec_-His_6_ formed a dimer, whereas His_6_-AphA_Ec_ formed a tetramer. Several crystal structures of AphA_Ec_ homologs were reported (22, 23, 24) and inspection of a published crystal structure of AphA_Ec_ homolog (PDB 2B82) suggested that a Ct His_6_ tag potentially leads to a steric clash between the neighboring two monomers (Fig. S1, *D* and *E*), destabilizing the tetrameric configuration. Both *E. coli* protein variants were therefore purified (Fig. S1*C*) and used to measure the binding affinity of cAMP *via* DRaCALA (Fig. 1*F*). Low-micromolar range K_d_ values were obtained for both proteins (4.4 ± 0.4 μM and 3.3 ± 0.4 μM for AphA_Ec_-His_6_ and His_6_-AphA_Ec_, respectively), suggesting a high-binding affinity of cAMP to AphA_Ec_ and that the His_6_ tags and multimeric states did not affect the binding of cAMP to AphA_Ec_ dramatically. As a control, the His_6_-tagged CRP_Ec_ from the ASKA library binds to cAMP with a K_d_ value of 53 ± 20 μM (Fig. 1*F*), similar to previously reported values (25, 26).

cAMP is chemically similar to AMP, a substrate of AphA_Ec_. We found, however, that AphA_Ec_ did not cleave cAMP (Fig. S1*F*), consistent with previous work (27). We then performed the DRaCALA competitive binding assay again with the purified AphA_Ec_ proteins and found that 100 μM of cAMP and cGMP, but not the substrates AMP, GMP, or other purine nucleotide diphosphates, triphosphates, were able to outcompete the binding of p^32^-α-cAMP by CRP_Ec_ and AphA_Ec_ (Fig. S1, *G*–*I*). Since low-micromolar range K_m_ values (3 and 15 μM,) of AMP and GMP were reported for AphA_Ec_ (27), the data appear to indicate that cAMP binds to a site different from the catalytic pocket of AphA_Ec_ (see below).

### cAMP competitively inhibits the acid phosphatase activity of AphA_Ec_

**Figure S2:**
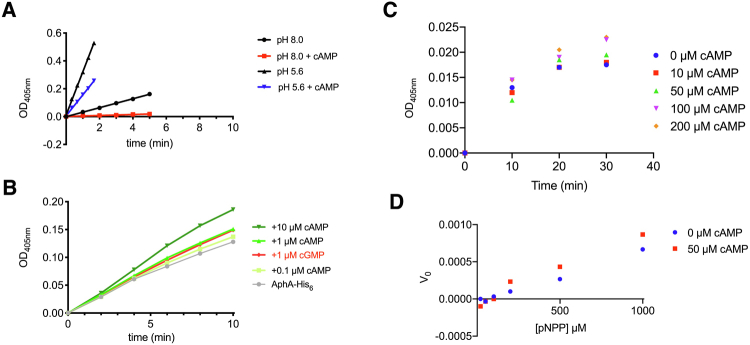
cAMP inhibits the catalytic activity of His_6_-AphA_Ec_ (*A*) but stimulates AphA_Ec_-His_6_ (*B*). The acid phosphatase assay was performed by using p-Nitrophenyl Phosphate (pNPP, 1 mM) as substrate and 15 nM of each AphA_Ec_ protein at pH 8 and 5.6 (see text and Method for details). The absorbance at 405 nm (*A*_405nm_) depicted over time illustrate the gradual accumulation of the reaction product pNP resulting from the cleavage of pNPP. cAMP (and cGMP) at various concentrations were added to assess their effects on the AphA_Ec_ activity. *C*, time course of AphA_Ec_-His_6_ cleaving pNPP in the absence and presence of varied concentrations of cAMP. *D*, Michaelis–Menten curves of AphA_Ec_-His_6_ cleaving pNPP in the absence and presence of 50 μM cAMP.

As a nonspecific acid phosphatase, AphA_Ec_ degrades many nucleotide monophosphates to generate nucleoside and orthophosphate (27). To understand the effect of cAMP on the AphA_Ec_ activity, we performed a phosphatase assay of AphA_Ec_ by using p-Nitrophenyl Phosphate (pNPP) as the substrate (27). Cleavage of pNPP releases a phosphate and pNP, a yellow chemical with a maximal absorption at 405 nm, which could be used to quantitate the reaction. His_6_-AphA_Ec_ was first tested at the reported optimal pH 5.6 and cAMP (100 μM) inhibited the catalytic activity of His_6_-AphA_Ec_ 2-fold (Fig. S2*A*). A similar assay was performed at a higher pH 8 and a 10-fold inhibition was observed despite the lower activity of His_6_-AphA_Ec_ (Fig. S2*A*). However, we found that cAMP stimulated the catalytic activity of AphA_Ec_-His_6_ at pH 5.6 in a dose-dependent manner (Fig. S2*B*). Despite the low fold of stimulation, the effect was highly reproducible (Fig. S2, *C* and *D*). Of note, the activity of AphA_Ec_-His_6_ was lower than His_6_-AphA_Ec_, and cGMP stimulated AphA_Ec_-His_6_ activity as well (Fig. S2*B*). Despite this observation, the dimeric configuration of AphA_Ec_-His_6_ is likely a nonnatural state (see below) and this artificial phenomenon was not studied further.

**Figure 2:**
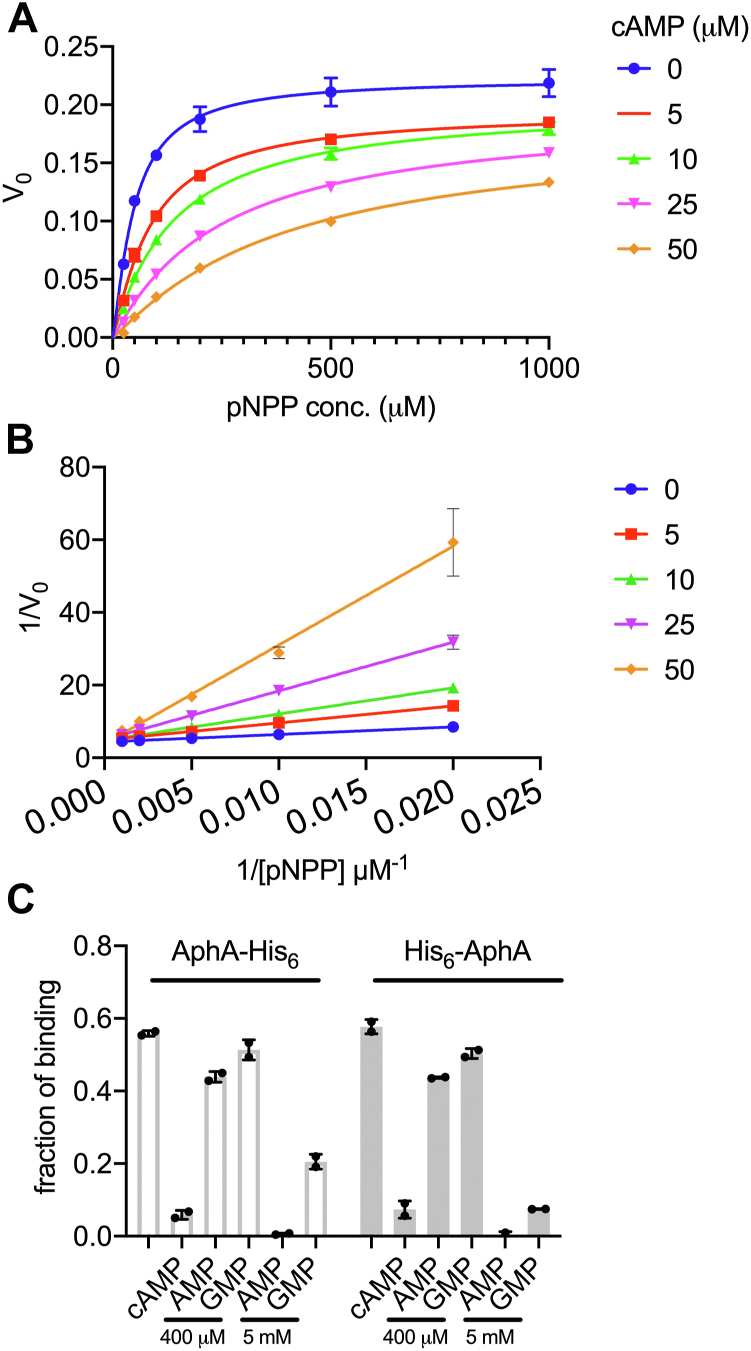
**cAMP competitively inhibits the phosphatase activity of AphA**_**Ec**_**of *Escherichia coli*.***A*, Michaelis–Menten curves and (*B*) the Lineweaver–Burk plot of tagless AphA_Ec_ cleaving p-Nitrophenyl Phosphate (pNPP) in the absence or presence of various concentrations of cAMP (see text and Method for details). Three biological replicates were performed, and the average and SD of mean are shown. *C*, quantitation of DRaCALA competition assay by using purified His_6_-AphA_Ec_ and AphA_Ec_-His_6_ proteins, without or with cold cAMP (100 μM), AMP (400 μM, 4 mM), and GMP (400 μM, 4 mM). Two biological replicates were performed, and the average and SD are shown.

**Figure S3:**
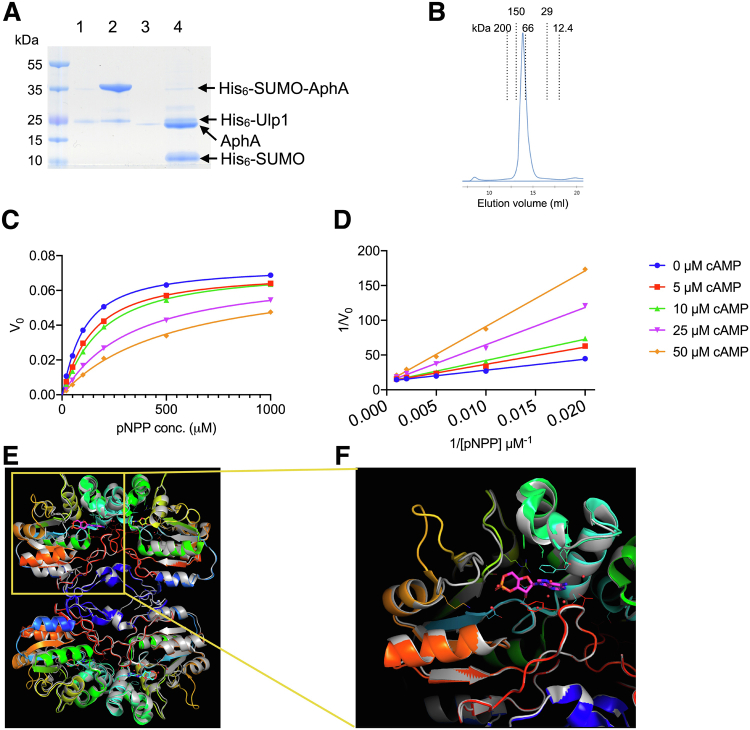
*A*, An SDS-PAGE gel test of noncleaved and cleaved His_6_-SUMO-AphA_Ec_ protein. 1, His_6_-Ulp1; 2, His_6_-SUMO-AphA_Ec_; 3, tagless AphA_Ec_ after cleavage; 4, the cleavage reaction mixture. *B*, FPLC profile of the cleaved tagless AphA_Ec_. The sizes of the standard proteins (kDa) are marked on the *top*. *C*, Michaelis–Menten curves and (*D*) the Lineweaver–Burk plot of His_6_-AphA_Ec_ cleaving pNPP in the absence or presence of cAMP. *E*, superposition of *Escherichia coli* AphA_Ec_ structure (2B82, *gray*) to the *Salmonella typhimurium* AphA in complex with cAMP (1U5Z, color). *F*, Zoom-in of the cAMP binding pockets in 1U5Z (*E*) and the superimposed 2B82.

Given the opposite effects of cAMP on the Nt and Ct His_6_–tagged AphA_Ec_, we constructed a tagless AphA_Ec_ to further clarify the regulatory effect of cAMP. To do this, we cloned AphA_Ec_ with a Nt His_6_-SUMO tag, which was cleaved off by using the SUMO-specific protease Ulp1 (His_6_-Ulp1). The SDS-PAGE gel (Fig. S3*A*) showed that the His_6_-SUMO tag was successfully cleaved off to generate a protein around 25 kDa, matching the expected tag-less AphA_Ec_. The SEC profile revealed a peak with the calculated size of 100 kDa, a tetrameric form of tag-less AphA_Ec_ (Fig. S3*B*). These data confirm that the tetrameric form is the natural state of AphA_Ec_. Subsequently, we performed the pNPP phosphatase assay by using the tag-less AphA_Ec_ and varied concentrations of cAMP (Fig. 2, *A* and *B*). The Michaelis–Menten curves were fitted with different models of inhibition. An allosteric sigmoidal fit was found to be the best, with the fitted Hill coefficient between 1 and 1.3. Moreover, fitting to the different models of inhibition and a Lineweaver–Burk plot (Fig. 2*B*) indicated that cAMP probably inhibits the catalytic activity of AphA_Ec_ in a competitive manner (K_i_ = 3.9 ± 0.3 μM). We then performed a kinetic study of the tetrameric His_6_-AphA_Ec_ and obtained very similar curves and K_i_ value (11 ± 0.6 μM) (Fig. S3, *C* and *D*). These data suggest that the Nt histidine tag does not greatly affect the activity of cAMP on AphA_Ec_. We therefore used the His_6_-AphA_Ec_ for the subsequent experiments.

The competitive inhibition of AphA_Ec_ activity contradicts the DRaCALA competitive binding assay (Fig. 1, *C*–*E*), which indicates an allosteric binding of cAMP. To study this further, we performed DRaCALA competitive binding assay by using even higher (400 μM) concentrations of AMP and GMP and observed a slight reduction of the binding fractions with AMP, but not GMP (Fig. 2*C*). We then used 5 mM of AMP and GMP. This time, the binding fractions dropped to nearly zero for AMP and to the similar level of 100 μM cAMP for GMP (Fig. 2*C*), consistent with the fact that AMP has a higher affinity to AphA_Ec_ than GMP (27).

A further search of AphA_Ec_ homologs in the PDB database found the AphA from *Salmonella typhimurium* (AphA_St_, 1Z5U), which was serendipitously crystalized in complex with cAMP (Fig. S3, *E* and *F*). AphA_St_ shares extensive amino acid sequence similarity (89% amino acid sequence identity) and striking structural similarity with AphA_Ec_ (2B82, Fig. S3, *E* and *F*, rmsd = 0.331). Superposition of both protein structures indicates that cAMP could bind to the catalytic site of AphA_Ec_. Altogether, these data suggest that cAMP binds to the active site of AphA_Ec_ and strongly inhibits its phosphatase activity.

### AphA from *H. influenza* Rd KW20 is competitively inhibited by cAMP

The authentic physiological function of AphA_Ec_ seems to cleave nucleotides to nucleosides used as the carbon source for *E. coli* (28), despite the report that AphA_Ec_ may bind to hemi-methylated DNA in *E. coli* (29). On the other hand, AphA homolog from *S. typhimurium* (AphA_St_) was reported to facilitate the uptake of NAD (30). Moreover, several pieces of evidence suggest that AphA in *Hemophilus influenzae* (AphA_Hi_) is functionally related to the natural competence in *H. influenzae*. (1) Nucleotides and nucleosides inhibit the natural competence of *H. influenzae* (4); (2) The promoter region of *aphA*_*Hi*_ is predicted to encode a binding site of PurR_Hi_, which regulates natural competence in *H. influenzae* (3). (3) Two positively charged surface areas exist and are conserved on AphA homolog proteins (Fig. 4, *A* and *B*, circulated). Therefore, we turned to study the potential function of AphA_Hi_ and cAMP in the natural competence of KW20.

**Figure 3:**
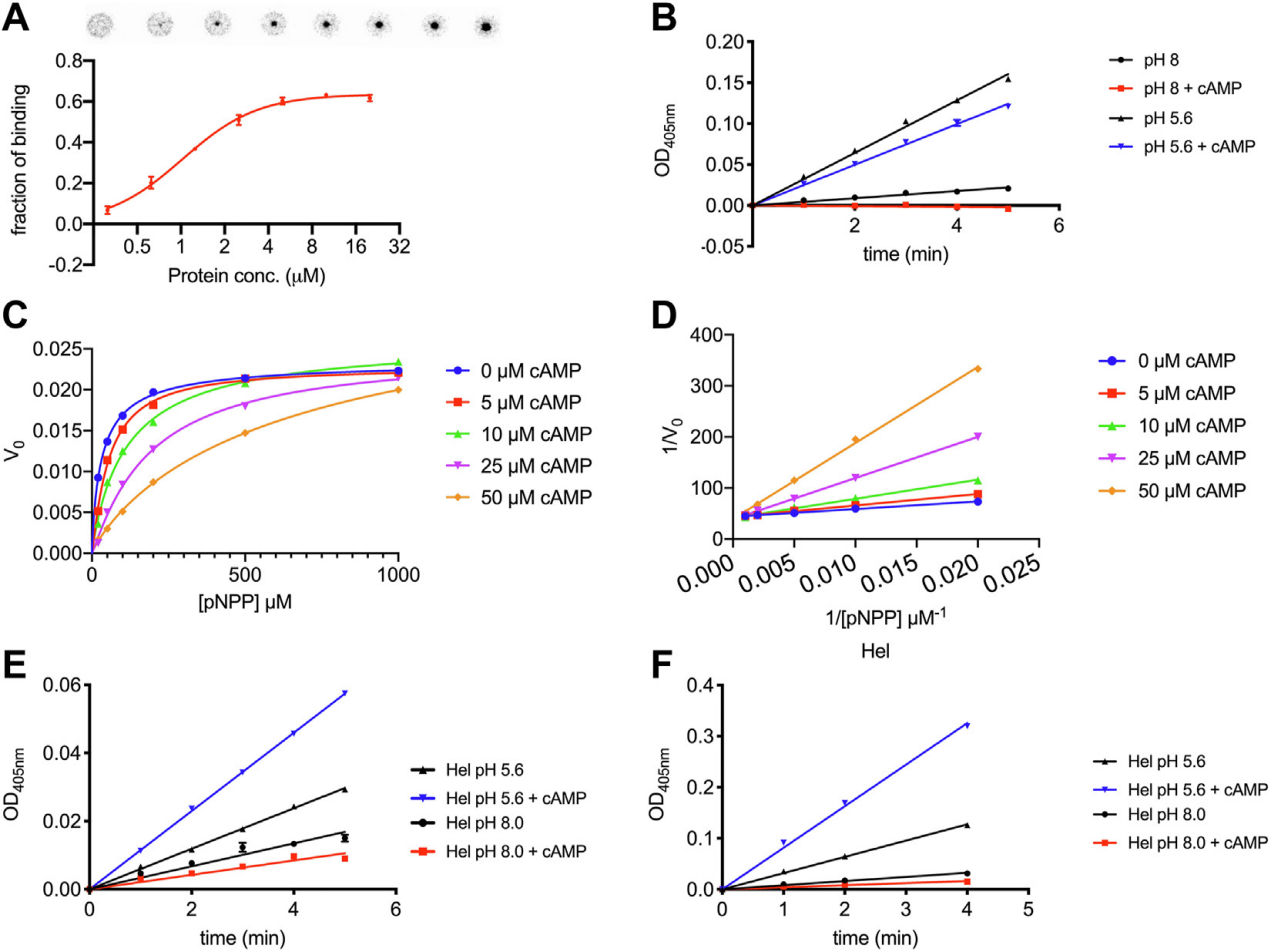
**cAMP strongly binds and competitively inhibits the AphA**_**Hi**_**from *Haemophilus influenzae*.***A*, DRaCALA assay to measure the binding affinity of His_6_-AphA_Hi_ to ^32^p-cAMP. Two biological replicates were performed, and the average and SD are shown. One set of representative DRaCALA spots was shown above the diagram. *B*, the acid phosphatase assay was performed by using p-nitrophenyl phosphate (pNPP, 1 mM) as substrate, 15 nM of His_6_-AphA_Hi_ protein at pH 8 and 5.6, without or with 100 μM cAMP (see text and Method for details). The absorbance at 405 nm (*A*_405nm_) depicted over time illustrate the gradual accumulation of the reaction product pNP resulting from the cleavage of pNPP. Three biological replicates were performed, and the average and SD of mean are plotted. *C*, Michaelis–Menten curves and (*D*) the Lineweaver–Burk plot of His_6_-AphA_Hi_ cleaving pNPP in the absence or presence of varied concentrations of cAMP. *E* and *F*, the acid phosphatase assay of the His_6_-Hel_Hi_ protein (*E*,15 nM) and a tagless Hel_Hi_ protein (*F*,15 nM) was performed by using pNPP (1 mM) as substrate, at pH 8 and 5.6, without or with 100 μM cAMP (similar as *panel B*, and see text and Method for details). At least two biological replicates were performed, and the average and SD are plotted.

Similar as the AphA_St_ (PDB, 1Z5U), AphA_Hi_ is a close homolog of AphA_Ec_ with 49% identity and 69% similarity at the primary sequence level. AphA_Hi_ is thus anticipated to have a similar structural fold, raising the possibility that cAMP also inhibits AphA_Hi_. To test this, we first purified the His_6_-AphA_Hi_ and tested its binding to cAMP *via* DRaCALA. His_6_-AphA_Hi_ binds to cAMP with a low-micromolar affinity (K_d_ = 1.03 ± 0.04 μM) (Fig. 3*A*). We then found that cAMP inhibits His_6_-AphA_Hi_ at both pH 5.6 and 8 (Fig. 3*B*). Further enzyme kinetic analysis showed that cAMP inhibits His_6_-AphA_Hi_ in a competitive manner (K_i_ = 6.9 ± 0.7 μM) (Fig. 3, *C* and *D*). This shows that cAMP strongly inhibits AphA_Hi_.

### His_6_-AphA_Hi_ does not bind to dsDNA or ssDNA

**Figure 4:**
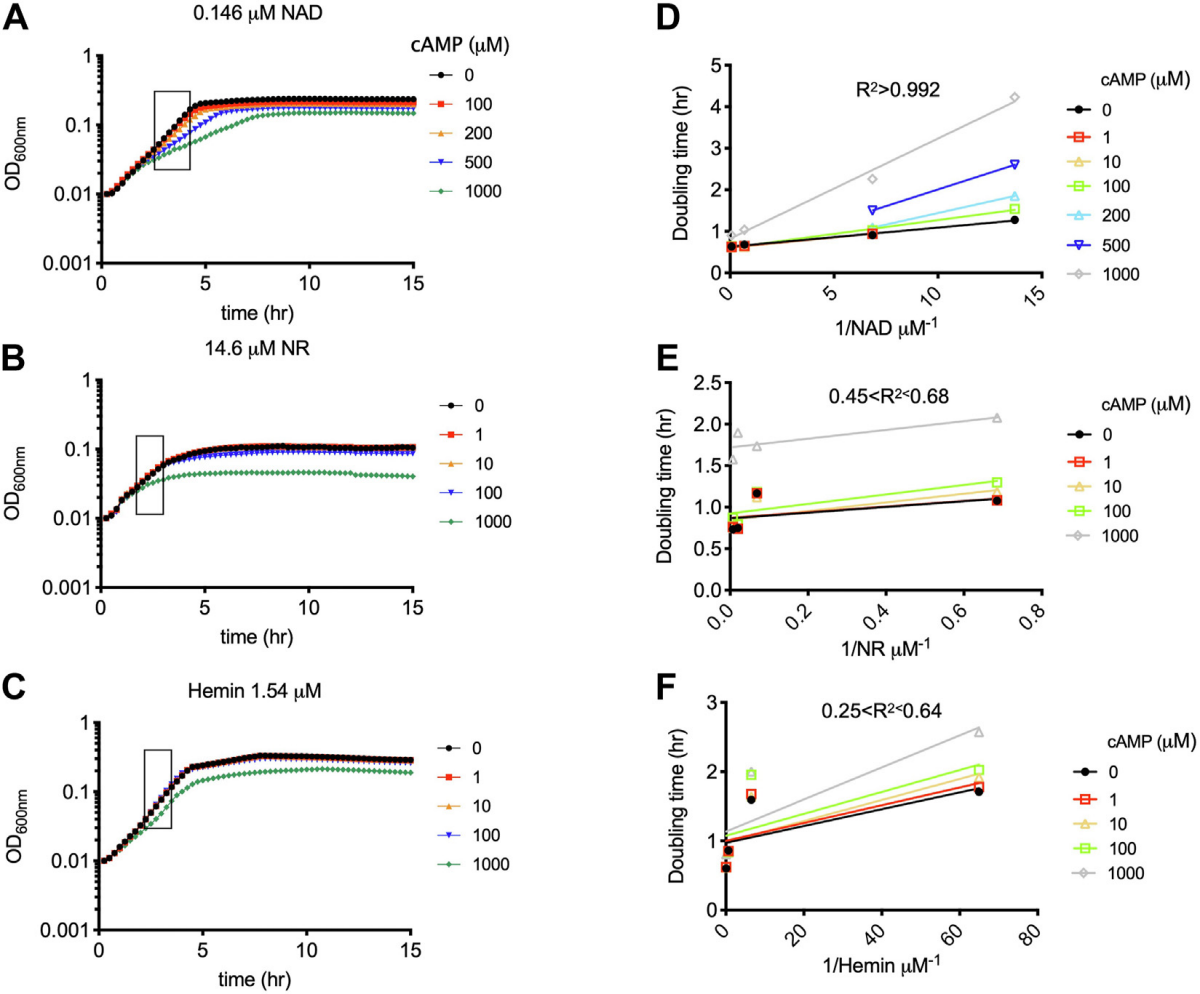
**cAMP competitively inhibits *Haemophilus influenzae* Rd KW20 growth rate in sBHI supplemented with NAD.***A*–*C*, representative growth curves of KW20 in sBHI medium supplemented with varied concentrations of the essential growth factors, that is, NAD (*A*, 0.146 μM), NR (*B*, 14.6 μM), and hemin (*C*, 1.54 μM) in the presence of different concentrations of cAMP (μM). The framed regions of exponential phase growth data were used to calculate the growthrates. See all growth curves in Fig. S6. Three biological replicates were performed. *D*–*F*, the double-reciprocal Lineweaver–Burk plot of the doubling time (*i.e.*, the reciprocal of growth rate, in hour) and the essential growth factors (in 1/[concentration of NAD/NR/hemin]) of wt *H. influenzae* were plotted. The average growth rate out of the three biological replicates (*A*–*C*) was used to calculate the doubling times. The R^2^ values of the linear fitting results are annotated above each diagram. NR, nicotinamide riboside; sBHI, brain heart infusion medium supplemented with hemin and NAD.

**Figure S4:**
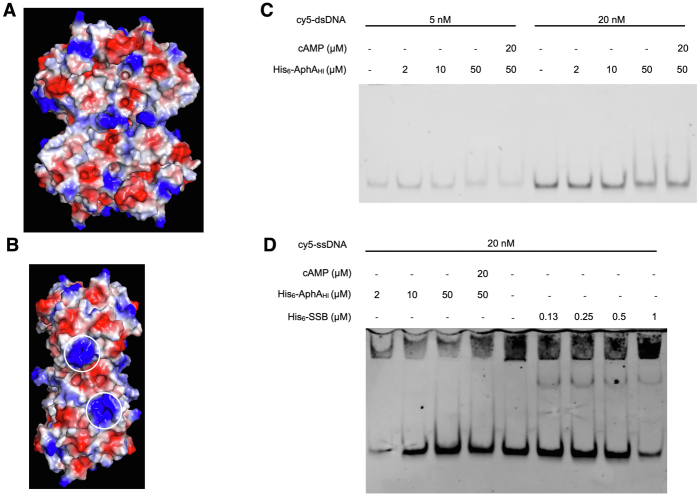
The front (*A*) and side (*B*) views of the surface charge of *Escherichia coli* AphA_Ec_ (PDB 2B82). *Blue* and *red* indicate positively and negatively charged areas, respectively. *C*, gel retardation assay testing the binding of His_6_-AphA_Hi_ (μM) to a 200-bp long dsDNA (5 and 20 nM) amplified from *Haemophilus influenzae* chromosome, which was labeled with cy5 at the 5’-ends. *D*, gel retardation assay similar as in *panel C*, except that the dsDNA was denatured ssDNA. The *E. coli* ssDNA-binding protein (His_6_-SSB) was used as positive control.

The periplasmic AphA_Hi_ may bind directly to imported DNA molecules given the conserved positive surface patches (Fig. 4, *A* and *B*). To test this, we amplified a 200-bp long DNA sequence from KW20, which contains a KW20 specific uptake sequence ACCGCACTT. Gel retardation assay showed that His_6_-AphA_Hi_ (up to 50 μM) did not shift the DNA band, regardless of the presence of cAMP (Fig. S4*C*). We then denatured the dsDNA to ssDNA (see method for preparation) and tested the binding again. As a positive control, we used the recombinant *E. coli* ssDNA-binding protein (His_6_-SSB_Ec_, P0AGE0). His_6_-SSB_Ec_ shifted the ssDNA from as low as 0.13 μM (Fig. S4*D*). However, we still did not see a shifted band with His_6_-AphA_Hi_ (up to 50 μM). These data suggest that His_6_-AphA_Hi_ does not directly bind to dsDNA or ssDNA.

### Δ*aphA*_*Hi*_ is not defective in the starvation medium M-IV induced competence

**Figure S5:**
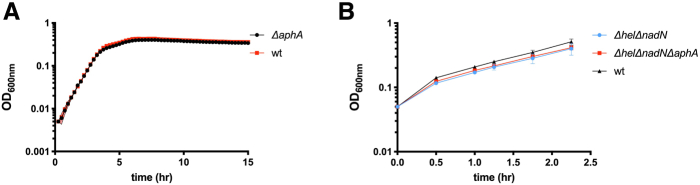
Growth curves (*A*_600nm_) of various strains of *Haemophilus influenzae* Rd KW20 in sBHI media supplemented with NAD (*A*) or nicotinamide riboside (NR) (*B*). Two biological replicates were performed, and the average and SD are shown.

To test directly if AphA_Hi_ is involved in KW20 competence, we constructed the *aphA*_*Hi*_ deletion strain Δ*aphA*_*Hi*_*::cat*, where *aphA*_*Hi*_ was replaced with a chloramphenicol resistance marker. Δ*aphA*_*Hi*_*::cat* grows similar as the WT KW20 in sBHI-rich medium (Fig. S5*A*). We then tested the competence phenotype under several conditions, that is, during growth into stationary phase in sBHI medium, M-IV starvation medium induced competence, and cAMP (1 mM) induced competence of log-phase sBHI culture. Nucleotides (and nucleosides) inhibit the natural competence in *H. influenzae* (4). Given the fact that AphA degrades nucleotides, we also tested the role of AphA_Hi_ in AMP-mediated inhibition of KW20 competence. However, there was no obvious difference in competence efficiency between wt KW20 and Δ*aphA*_*Hi*_*::cat* strains under all the tested conditions (data not shown).

### cAMP affects the catalytic activity of Hel_Hi_ in a pH-dependent manner

The current model of nucleotide-mediated inhibition of KW20 competence proposes that extracellular nucleotides are degraded to nucleosides, which enter the cytosol to inhibit the translation of the master regulator of competence, Sxy_Hi_ (3). Besides AphA_Hi_, KW20 encodes two additional phosphatases, the periplasmic NadN (NadN_Hi_) and the outer membrane anchored Hel (Hel_Hi_, e(P4)). Hel_Hi_ is a close structural homolog of AphA (31), and dephosphorylates NADP, NMN, and nucleotides (32). NadN_Hi_ first degrades NAD to NMN and AMP, and then both NMN and AMP are further dephosphorylated by NadN_Hi_ into NR and adenosine, which traverse the inner membrane (12). Therefore, NadN_Hi_ and Hel_Hi_ are functionally redundant with AphA_Hi_ regarding dephosphorylation of the compounds mentioned and thereby potentially the inhibition of competence as well.

To test if cAMP also competitively inhibits NadN_Hi_ and Hel_Hi_, we purified His_6_-NadN_Hi_ and His_6_-Hel_Hi,_ and performed the phosphatase assay with pNPP and cAMP. cAMP inhibited the phosphatase activity of His_6_-Hel_Hi_ at pH 8, but surprisingly stimulated it at pH 5.6 (Fig. 3*E*). To rule out the potential effect of the histidine tag, we purified a tagless Hel_Hi_ as above (see Experimental procedures). However, the pH-dependent effect of cAMP still holds for the tagless Hel_Hi_ (Fig. 3*F*). The physiological niche of KW20, mucus, has a pH range of 6 to 7, and Hel_Hi_ localizes on either the inward or outward side of the outer membrane. It thus remains plausible that cAMP inhibits the phosphatase activity of His_6_-Hel_Hi_ under physiological conditions.

### cAMP competitively reduces the growth-rate of KW20 in sBHI supplemented with NAD

The recombinant His_6_-NadN_Hi_ proteins purified from *E. coli* BL21 DE3 did not show any activity toward pNPP on our hands (data not shown). We thus turned to a whole-cell based approach. NadN_Hi_ is an essential protein for KW20 growth (14) because KW20 cannot synthesize NAD and NadN_Hi_ degrades exogenous NAD to NMN and further to NR, which then traverses the inner membrane of KW20 *via* PnuC (12, 13). Inside KW20 cells, NR is converted back to NAD used for essential metabolic processes. Therefore, an exponentially growing KW20 cell is expected to contain a fixed amount of intracellular NAD (iNAD) per cell volume unit (diNAD/dV). The increase of iNAD per time (diNAD/dt) reflects the reaction speeds of firstly NadN_Hi_ and then enzymes of subsequent steps. diNAD/dt is also proportional to the increase of cell size, which can be measured by OD_600nm_ change per time (dOD_600nm_/dt, *i.e.*, the growth rate). To test if cAMP inhibits the NadN_Hi_ function, one can then vary the concentrations of reaction substrate, that is, exogenous NAD in the sBHI medium, and measure cell growth rates, that is, the reaction product, in the presence of varied concentrations of the inhibitor, cAMP. By analogy to classic biochemical reactions, a double-reciprocal Lineweaver–Burk plot of the cell growth rate and the substrate concentration can indicate if cAMP inhibits NadN_Hi_ function in a competitive manner. To control for the subsequent reactions after NadN_Hi_ cleaves NAD and NMN, for example, the transfer of NR inside KW20 and conversion of NR back to iNAD, we performed the same growth experiments by supplementing NR, instead of NAD, in sBHI medium. As a further control, the same experiments were repeated with another essential growth factor hemin, whose utilization is independent of NadN_Hi_. At last, since a high concentration of cAMP (1–10 mM) induces competence and inhibits the growth of KW20, we tested a much lower range of cAMP (from 1 to 500 μM).

**Figure S6:**
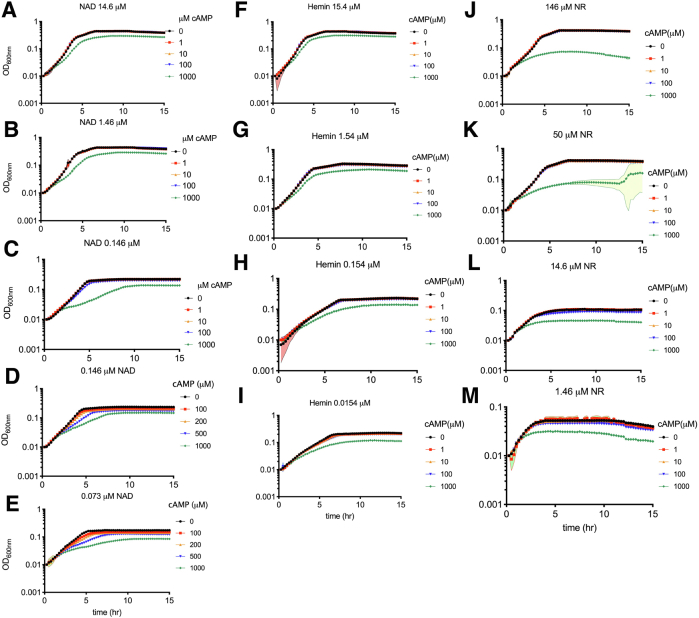
Growth curves (*A*_600nm_) of the *Haemophilus influenzae* Rd KW20 in sBHI medium supplemented with varied concentrations of NAD (*A*–*E*), nicotinamide riboside (NR) (*F*–*I*), and hemin (*J*–*M*) in the presence of differential concentrations of cAMP. Three biological replicates were performed, and the average and SD of mean are shown.

Representative growth curves and the exponentially growing phases are shown in Figure 4, *A*–*C* and S6. We then performed a double-reciprocal Lineweaver–Burk plot of the growth rate (*i.e.*, the doubling time, in hour per cell division) and substrate concentrations (*i.e.*, in 1/[substrate concentration, μM^−1^]) (Fig. 4, *D* and *E*). Figure 4*D* shows the characteristic straight lines converging to the *y*-axis (R^2^ > 0.992) and indicates that cAMP reduces the growth-rate in a competitive manner when NAD was studied. As controls, when NAD was replaced with NR (Fig. 4*E*) or when hemin was assayed (Fig. 4*F*), the curves were poorly fitted (R^2^ between 0.25 and 0.68) and thus inconsistent with a competitive inhibition model. These data suggest that cAMP competitively inhibits the function of NadN_Hi_ in the cleavage of NAD to NMN and NR.

### The triple mutant Δ*nadN*_*Hi*_Δ*hel*_*Hi*_Δ*aphA*_*Hi*_ is refractory to the inhibitory effect of nucleotides on M-IV induced competence

**Figure 5:**
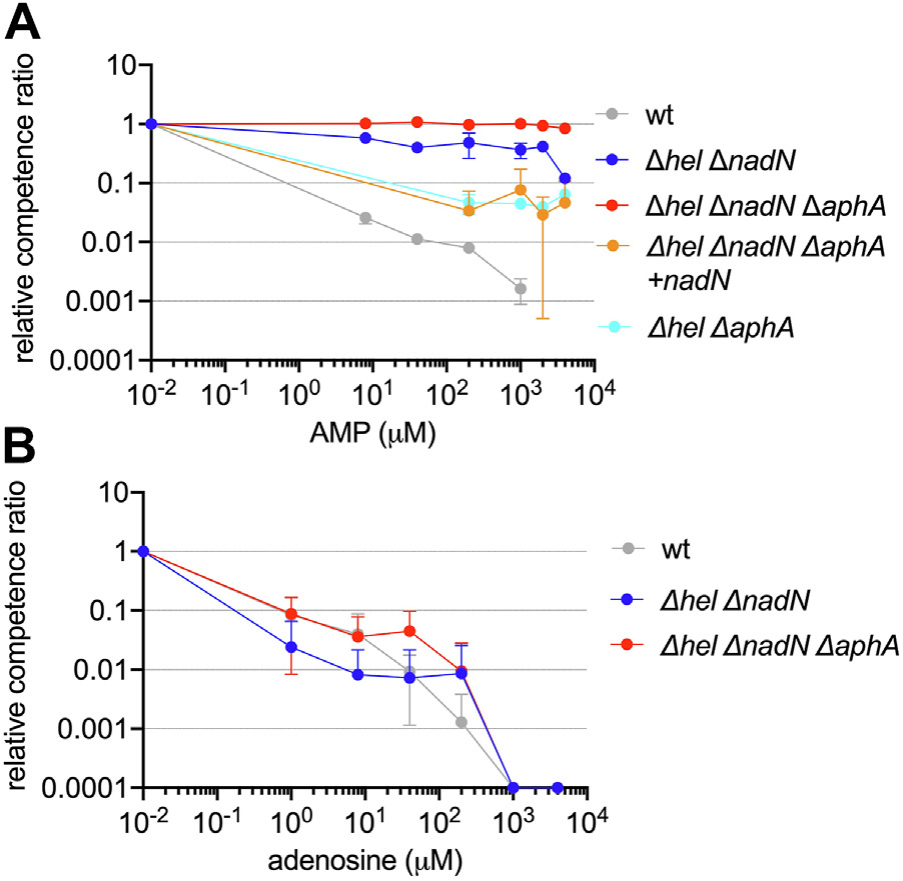
**The triple mutant Δ*hel***_***Hi***_**Δ*nadN***_***Hi***_**Δ*aphA***_***Hi***_**is refractory to the inhibitory effect of nucleotide on competence development in *Haemophilus influenzae* Rd KW20.***A*, relevant competence ratios of wt, Δ*hel*_*Hi*_Δ*nadN*_*Hi*_, Δ*hel*_*Hi*_Δ*aphA*_*Hi*_*,* Δ*hel*_*Hi*_Δ*nadN*_*Hi*_Δ*aphA*_*Hi*_*,* and the complemented strain Δ*hel*_*Hi*_Δ*nadN*_*Hi*_Δ*aphA*_*Hi*_ + *nadN*_*Hi*_ in the presence of varied AMP concentration. The competence level of wt KW20 in the absence of AMP was used to normalize the competence of other strains. To plot the data in the absence of AMP (*i.e.*, 0 μM), an arbitrary value, 0.01 μM, was used. At least two biological replicates were performed, and the average and SD are shown. *B*, similar as *A*, except that the competence was determined in the presence of varied concentrations of adenosine. Five biological replicates were performed, and the average and SD are shown.

The above data showed that cAMP inhibits the phosphatase activities of AphA_Hi_, NadN_Hi_, and Hel_Hi_. Given their redundant activities, we proposed that AphA_Hi_, NadN_Hi_, and Hel_Hi_ altogether degrade nucleotides to nucleosides, to inhibit KW20 competence development. To test, we first found that the growth dynamics are similar in sBHI supplemented with NR (sBHI+NR) for wt, Δ*hel*_*Hi*_Δ*nadN*_*Hi*_ and Δ*hel*_*Hi*_Δ*nadN*_*Hi*_Δ*aphA*_*Hi*_ mutants, confirming that the main function of NadN_Hi_ is for utilizing NAD (Fig. S5*B*). The three strains were grown in sBHI+NR broth to log phase and shifted to M-IV starvation medium to induce competence. Fifteen minutes after the shift, various concentrations of AMP were added. As shown in Figure 5*A*, AMP from as low as 10 μM reduced the M-IV induced competence by ca. two orders of magnitude in wt KW20. The triple mutant was completely refractory to the inhibitory effect of AMP (up to 4 mM tested). Importantly, genetic complementation of the triple mutant with *nadN*_*Hi*_ restored an intermediate phenotype, like other double deletion mutants Δ*hel*_*Hi*_Δ*nadN*_*Hi*_ and Δ*hel*_*Hi*_Δ*aphA*_*Hi*_ (Fig. 5*A*). These data suggest that AphA_Hi_, NadN_Hi_ and Hel_Hi_ all contribute to the inhibitory effect of AMP on KW20 competence likely by cleaving AMP to adenosine. Consistently, competence of the double and triple mutants was completely inhibited by adenosine (Fig. 5*B*).

**Figure S7:**
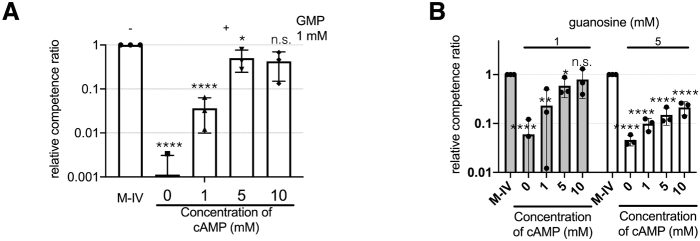
The M-IV induced competence development in *Haemophilus influenzae* Rd KW20 is affected by cAMP, nucleotides, and nucleosides. *A* and *B*, relevant competence ratios of wt KW20 in the absence and presence of 1 mM GMP (*A*), 1 or 5 mM guanosine (*B*), and varied concentrations of cAMP. Three biological replicates were performed, and the average and SD of mean are shown. Unpaired *t* test was used to compare the relative competence ratio in the presence of GMP and varied cAMP with that in their absence. ∗, *p* ≤ 0.05; ∗∗, *p* ≤ 0.01; ∗∗∗∗, *p* ≤ 0.0001; n.s., not significant.

Since cAMP competitively inhibits the activities of the three phosphatases, we added cAMP (1–10 mM) in the assay to test if cAMP counteracts AMP in KW20 competence development. We observed that the AMP inhibited competence of wt KW20 was not restored by cAMP, consistent with previous report (4) (data not shown; see discussion below). Instead, we tested if cAMP restores the competence inhibited by GMP (1 mM), which has a weaker ability than AMP to outcompete the binding of ^32^p-cAMP to AphA (Fig. 2*C*) and to inhibit competence (4). We found that cAMP restored the inhibited competence in a dose-dependent manner (Fig. S7*A*), consistent with the previous report (4). Finally, we showed that cAMP in a dose-dependent manner partially restored the KW20 competence inhibited by 1 mM guanosine, and the restoration was worse with 5 mM guanosine (Fig. S7*B*). Taken together, these data demonstrate the key role of AphA_Hi_, NadN_Hi_, and Hel_Hi_ in regulating *H. influenzae* KW20 competence with regard to cAMP and purine nucleotides.

## Discussion

**Figure 6:**
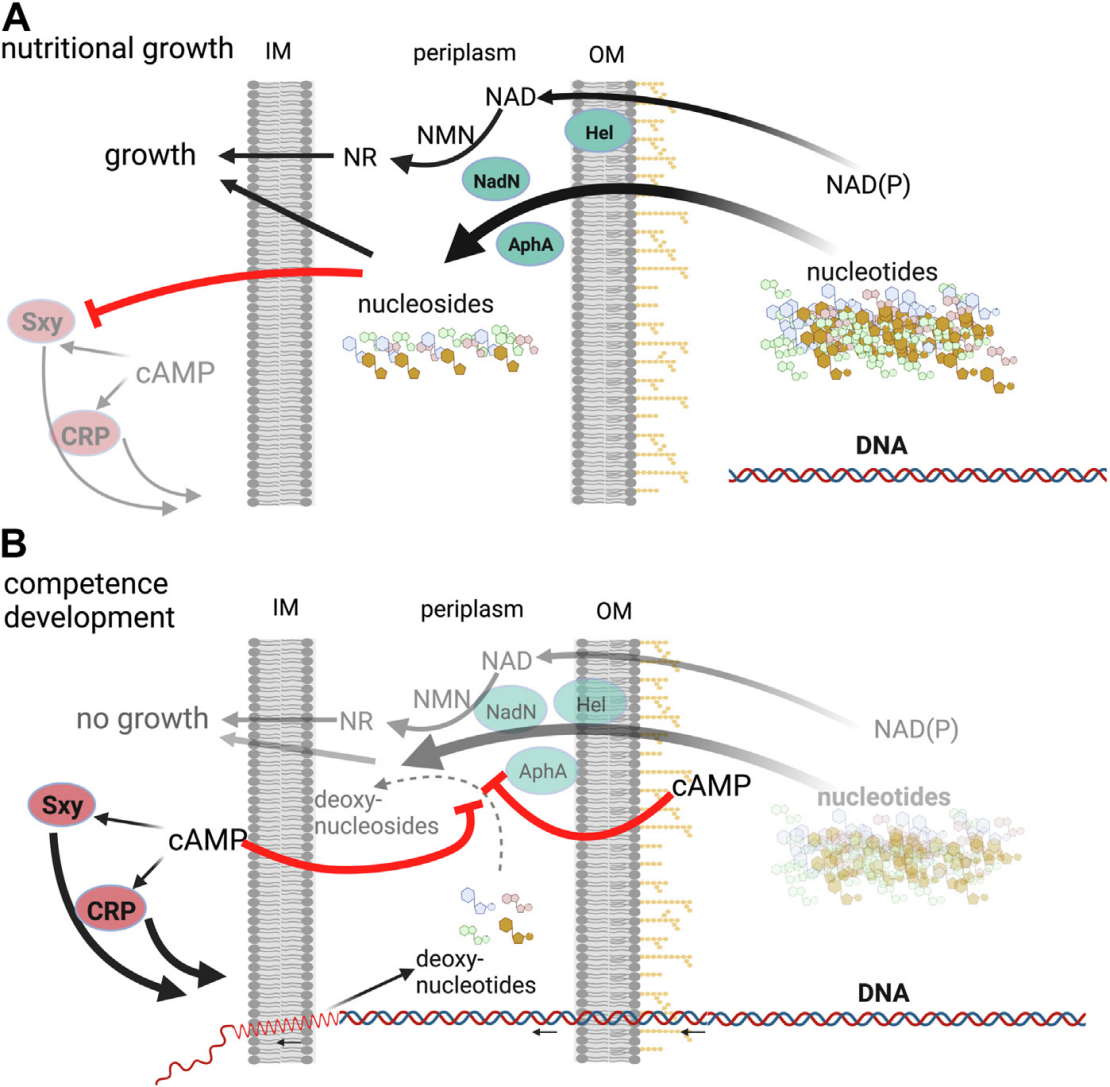
**Model of the periplasmic phosphatases in coupling the cell growth with competence development in *Haemophilus influenzae* Rd KW20.***A*, under nutritional condition, plenty of external (both purine and pyrimidine) nucleotides enter the periplasm where they are degraded by AphA_Hi_, Hel_Hi_, and NadN_Hi_ to nucleosides, which traverse the inner membrane (IM) to the cytosol to support cell growth and inhibit the production of Sxy_Hi_, consequently the competence development. Degradation of NAD(P) to NR by Hel_Hi_ and NadN_Hi_ provides the essential growth factor V for KW20. *B*, with the carbon source exhausted, cAMP is produced by KW20. On the one hand, cAMP competitively binds to AphA_Hi_, NadN_Hi_, Hel_Hi_, and inhibits their activities, slowing the generation of essential growth factors NR and pyrimidines and thus cell growth rate. Eventually, purine nucleotides are sufficiently depleted and the master competence regulator Sxy_Hi_ is produced to stimulate the gene expression required for competence development and DNA uptake. The conversion of deoxynucleotides from degrading one DNA chain during DNA uptake to deoxynucleosides is anticipated to be inhibited by cAMP as well. (Created with BioRender.com). NR, nicotinamide riboside.

Bacterial competence development is often characterized by a simultaneously inhibited cell growth (2), which results from the exhaustion of a key nutrient, typically a carbon source that consequently stimulates the production of cAMP. In *H*. *influenzae* KW20, competence is inhibited by purine nucleotides, AMP and GMP, and the corresponding nucleosides, but not the nucleobases (4). KW20 is a fastidious bacterium requiring several essential factors, including NAD, hemin, and pyrimidines, to grow. How KW20 perceives and coordinates these nutritional signals with competence development remains incompletely understood. In this study, we found that cAMP competitively inhibits the periplasmic nonspecific phosphatases AphA_Hi_ and Hel_Hi_ in KW20. Importantly, we showed that cAMP competitively inhibits the KW20 growth-rate in sBHI medium supplemented with NAD, but not NR, strongly suggesting that cAMP inhibits NadN_Hi_. Since NadN_Hi_ also degrades various nucleotides, it is anticipated that cAMP binds to the active site and competitively inhibits its activity. Moreover, only the triple deletion mutant Δ*hel*_*Hi*_Δ*nadN*_*Hi*_Δ*aphA*_*Hi*_ was refractive to the inhibitory effect of AMP on M-IV induced competence of KW20, consistent with the redundant activities of the three periplasmic phosphatases in cleaving nucleotides to nucleosides. We therefore propose a model of cAMP and the three phosphatases in coordinating KW20 cell growth and competence development (Fig. 6).

### cAMP inhibits AphA_Hi_, NadN_Hi_, and Hel_Hi_ to coordinate nutritional growth with competence development in *H. influenzae* Rd KW20

Under growth conditions with plenty of carbon sources, NAD(P), and nucleotides (Fig. 6*A*), Hel_Hi_ cleaves NADP to NAD (12, 15), and NadN_Hi_ cleaves NAD to NMN and NR, providing the essential factor V for KW20 growth (32). AphA_Hi_ might contribute to NR generation given its phosphatase activity. However, neither Hel_HI_ nor AphA_Hi_ is essential for KW20 growth using NAD (Fig. 5*A*; (14)). Consistently, the double Δ*hel*_*Hi*_Δ*nadN*_*Hi*_ and triple Δ*hel*_*Hi*_Δ*nadN*_*Hi*_Δ*aphA*_*Hi*_ mutants showed growth patterns similar to wt strain in the sBHI medium supplemented with NR (Fig. S5*B*). Besides NAD, all three proteins degrade various nucleotides to nucleosides (27, 32), providing both the essential pyrimidines, and carbon and energy sources for cell growth.

Upon carbon starvation (Fig. 6*B*), *H. influenzae* produces cAMP. However, whether the *sxy*_*Hi*_ mRNA is translated to produce Sxy_Hi_ depends on the exogenous nucleotides (3). Given the competitive inhibition of the phosphatase activities of Hel_Hi_, NadN_Hi_, and AphA_Hi_ by cAMP, we suggest that under starvation, cAMP and nucleotides compete for binding to the active sites of the three enzymes (Figs. 3 and 4). Consistently, cAMP binds to AphA_Hi_ with a low-micromolar affinity (K_d_ = 1.03 ± 0.04 μM, Fig. 3*A*) and a K_i_ of 6.9 ± 0.7 μM (Fig. 3*C*); furthermore, low micromolar k_m_ values (3 and 15 μM, respectively) of AMP and GMP to AphA were reported (27). Moreover, 100 μM of cAMP outperformed 400 μM of AMP/GMP and behaved similar as 5 mM of AMP/GMP, in competing away the bound radio labeled cAMP (Fig. 2*C*). Therefore, a competitive mechanism is conceivable and physiologically relevant, given the reported micromolar range of intracellular cAMP (from 1–10 μM (33) to 20–180 μM (34)) and the notion that the majority of synthesized cAMP is excreted outside cells (35, 36). Despite the inhibitory effect of cAMP, the excess nucleotides, especially pyrimidine nucleotides, are still gradually degraded by the three enzymes and utilized by KW20 for (slowed) cell growth (Fig. 4). Meanwhile, cAMP (of 1–500 μM range) competitively inhibits NadN_Hi_ from providing NR that is essential for growth (Fig. 4), thereby gradually reducing the growth rate. Additionally, the produced nucleosides inhibit the translation of *sxy*_*Hi*_ (3). A dynamic equilibrium is reached when the exogenous nucleotides are not of a high enough concentration to compete with cAMP. This concentration threshold is expected to be lower for AMP than for GMP, given the higher affinity of AMP to AphA (Fig. 2*C*) and the fact that cAMP cannot overcome the inhibition of competence by AMP ((3), data not shown) but by GMP (Fig. 5). Thereafter, further nucleosides are not provided and translation of *sxy*_*Hi*_ is derepressed to initiate competence development. Consistently, the triple mutant was completely refractory to the inhibitory effect of AMP (Fig. 5*A*) but not of the nucleoside adenosine (Fig. 5*B*). Moreover, elevated levels of cAMP restored the competence inhibited by 1 mM GMP, but not so well for 1 and 5 mM guanosine (Fig. S7). Of note, exogenous cAMP (mM) may be degraded by the periplasmic cAMP–specific phosphodiesterase CpdA (K_m_ ca. 0.5 mM) (36), producing AMP that could further inhibit competence. This may explain why cAMP was unable to restore competence inhibited by AMP (observed in both (3) and this study). It is thus obvious that the competitive inhibitory effect of cAMP on the three enzymes ensures a second layer of control such that only when the external nucleotides are sufficiently depleted the competence program commences. The competitive feature is likely a key mechanism of KW20 to gauge the concentrations of cAMP and nucleotides for coordinating cell growth and competence development. As discussed, KW20 does so likely because it is auxotrophic to NAD and pyrimidine, and additionally, nucleotides provide both phosphate and carbon sources. Nucleotides are energetically expensive to synthesize from the beginning (*de novo* pathway); thus the usage of external nucleotides saves significant amounts of energy that may enhance ecological fitness and virulence of Bacteria. Consistently, in many other bacteria, such as *E. coli*, the presence of nucleotides and nucleobases prevents the expression of genes involved in the *de novo* nucleotide biosynthesis *via* the PurR and CytR repressors (37, 38).

After the extracellular nucleotide pool is sufficiently depleted and Sxy_Hi_ protein is produced, the competence regulon is fully induced, including the machinery to take up extracellular DNA (10). The dsDNA then traverses the outer membrane into the periplasm, where one strand of the dsDNA enters the cytosol, and the other strand is degraded to deoxynucleotides and released in the periplasmic space. AphA_Hi_ has similar activities toward deoxynucleotides as nucleotides (27) and, most likely, both NadN_Hi_ and Hel_Hi_ degrade deoxynucleotides as well. The accumulated deoxynucleotides potentially bind to and compete for the binding site of cAMP on the three enzymes, thereby producing deoxynucleosides and phosphates. Although it is tempting to assume that these deoxynucleosides may enter the cytosol and feedback inhibit competence, it was shown before that deoxynucleosides do not inhibit competence induction of KW20 (4). Consistently, nucleotides only inhibit the early, but not the late, phase of competence induction, that is, the translation of Sxy protein (3).

### Nucleotides regulate the competence development of other bacteria

Purine nucleotides inhibit the competence development of Pasteurellacean strains closely related to KW20, that is, *Actinobacillus pleuropneumoniae* and *Actinobacillus suis* (3). Both bacteria encode homologs of the essential competence proteins and their competences depend on Sxy and CRP-cAMP. Additionally, both strains encode homologs of NadN_Hi_ (26–27% identity, 42–43% similarity), Hel_Hi_ (65% identity, 79% similarity), and AphA_Hi_ (48.4% identity, 64% similarity), except that *A. pleuropneumoniae* lacks an AphA_Hi_ homolog. Therefore, we propose that cAMP also competitively inhibits the phosphatase activities of these enzymes, to couple purine nucleotide depletion with competence development.

Nucleotides also inhibit the competence of *Vibrio cholerae* (39). Natural competence in *V. cholerae* requires two signals, that is, the presence of chitin and high cell density, and depends on cAMP, CRP, and TfoX (a Sxy homolog). Recently, it was found that deletion of *cytR*_Vc_ reduced the *V. cholerae* competence to the level similar to that seen in the presence of external nucleoside cytidine (100 mM) (39). In *E. coli*, CytR represses the expression of a set of genes involved in scavenging external nucleosides (40, 41), and cytidine derepresses their expressions by direct binding to CytR_Ec_. Currently, it is believed that CytR_Vc_ binds to cytidine to derepress an unknown factor that represses the expression of genes involved in natural competence development. However, besides cytidine, neither CMP nor purine nucleotides were tested in the competence development of *V. cholerae* (39). Further, it is unknown whether cAMP restores the decreased competence by cytidine. *V. cholerae* encodes neither AphA_Hi_ nor Hel_Hi_ homologs, but a homolog of NadN_Hi_, that is, UshA_Vc_ (37% identity, 52% similarity). Besides, *V. cholerae* encodes an alkaline phosphatase PhoX and CpdB_Vc_, which are homologs of PhoA_Ec_ and the cAMP-specific phosphodiesterase CpdB_Ec_ protein, respectively. UshA_Vc_, PhoX, and CpdB_Vc_ are important for *V. cholerae* to use nucleotides and DNA as phosphate sources (42). Given the chemical similarity of cAMP to nucleotides, we propose that cAMP inhibits the phosphatase activities of these proteins, serving as a possible mechanism that couples competence development with nucleotide scavenging. Another unique aspect of *V. cholerae* is that deoxycytidine also inhibits the *V. cholerae* competence (39), while deoxynucleosides do not inhibit KW20 competence (4).

The differential use of purine and pyrimidine nucleotides in regulating bacterial competence in KW20 and *V. cholerae* elicits some interesting questions regarding how the natural competence systems evolved. The competence systems seem to depend on both the native niches and the metabolic features of the specific bacterium. KW20 cannot synthesize NAD or pyrimidines (3). Therefore, the three proteins, NadN_Hi_, Hel_Hi_, and AphA_Hi_, likely play a crucial function in generating NR and pyrimidine nucleosides from mucus, the native niche of KW20 (11). The absolute requirement for pyrimidine and NAD for KW20 growth likely tuned the three acid phosphatases to constantly scavenging and depleting NAD and pyrimidines before the competence system is activated to take up external DNA as carbon, pyrimidine, and energy sources. Despite this, it is surprising that purine but not pyrimidine suppresses the competence of KW20, although KW20 has no CytR homolog but a PurR homolog. The scenario is even less clear for *V. cholerae.* The NAD and both purine and pyrimidine nucleotide synthesis pathways are complete, except for the incomplete pathway of deoxythymidine triphosphate synthesis*.* Thymine is therefore required for *V. cholerae* growth. *V. cholerae* produces exonucleases outside cells to degrade DNA and release nucleotides (43, 44). Among these, deoxythymidine monophosphate may be further degraded by periplasmic phosphatases to thymidine, which enters the cytosol and is used for DNA synthesis. Neither purine nor other pyrimidine nucleosides were tested in *V. cholerae*, which remains an unclear picture, warranting further analysis in this important human pathogen.

## Experimental procedures

### Bacterial strains, growth, media, and antibiotics

The strains and primers used in this project were listed in the Tables S1 and S2, respectively. Antibiotics used for the specific strains are listed in Table S1. The *E. coli* K-12 MG1655 is the WTstrain used. Lysogeny broth (LB, containing 10 g tryptone (Oxoid), 5 g yeast extract (Oxoid), and 10 g NaCl (SIGMA) per liter of distilled water) was the primary medium used for *E. coli* growth. For competence assays *H. influenzae* Rd KW20 was the WT used. A brain heart infusion medium (Becton and Dickinson) supplemented with 15.4 μM hemin and 14.6 μM NAD (sBHI) was the rich medium for this strain. For competence induction, M-IV medium was prepared as described before (45). LB and sBHI agar plates contained 1.5% agar (Difco).

### Competence assays

Natural competence in *H. influenzae* cells was performed as in (3). Briefly, competence was induced by transferring early log-phase cells (OD_600_ ≈ 0.2) grown in sBHI to the M-IV starvation medium and incubated for 1 h at 100 rpm, 37 °C. If cAMP was included in the assay, it was added at the onset of competence induction. When testing for the repressive effect of nucleotide precursors on competence development, this compound was added 15 min into competence induction. Cells were next incubated with 1 μg/ml purified chromosomal *H. influenzae* DNA encoding novobiocin resistance (from strain YZ1080) for 1 h and were then serial diluted in brain heart infusion medium and spotted on sBHI plates with or without 2.5 μg/ml novobiocin (SIGMA). Competence development was measured by dividing the novobiocin resistant CFU by the total CFU. Note that the YZ1080 strain was transformed with the MAP7 DNA (45) containing seven antibiotic markers in KW20 genomic DNA and selected on sBHI supplemented with 2.5 μg/ml novobiocin.

### Deletion of aphA_Hi_, hel_Hi_, and nadN_Hi_ in *H. influenzae* Rd KW20

For deletion of *aphA*_*Hi*_, *hel*_*Hi*_, and *nadN*_*Hi*_, the natural competence of *H. influenzae* was utilized to replace each of these genes with an antibiotic marker. Each of the antibiotic marker genes are proceded with the promoter sequence (TAAATTGAACTTTTTTCTTCATCAGAACTCAAAAACAACGTTCTCTGCCTAATTGAATTGGGCAGAGAAAATATTAAACCCATCATTTAATTAAGGATATTTATCAA) from a constitutively expressed KW20 gene *omp26*. Then, ca. 1000 bp homologous DNA sequences upstream and downstream of the *aphA*_*Hi*_*, hel*_*Hi*_*, nadN*_*Hi*_ genes were fused to the antibiotic marker *via* overlap PCR, to facilitate the recombination between the endogenous genes and the antibiotic marker sequences. As an example, for deletion of *aphA*_*Hi*_, primers DaphA-1 (*AACGGCGCGCAATTTCAGTTTTACC*) and DaphA-2 (CTGATGAAGAAAAAAGTTCAATTTA*tgcctttcctcacaaacgctgatt*) were used to PCR amplify the upstream sequence of *aphA* gene, and primers DaphA-3 and DaphA-4 were used to amplify the *omp26* promoter DNA, and primers DaphA-5 and DaphA-6 were used to amplify the chloramphenicol resistant marker for pWRG99 plasmid (46), and primers DaphA-7 and DaphA-8 were used to amplify the downstream sequence of *aphA*_*Hi*_ gene. The four DNA sequences were then fused together *via* overlap PCR to obtain the DNA to be fed to the competent KW20 cells and selected on sBHI media supplemented with chloramphenicol 4 μg/ml. Similarly, for deletion of *hel*_*Hi*_, primers pYZ653 and pYZ654, pYZ655 and pYZ656, pYZ657 and pYZ658, and pYZ659 and pYZ660 were used to amplify the corresponding four DNA fragments that were fused together *via* overlap PCR. The same approach was done for deletion of *nadN*_*Hi*_ but using primers pYZ661 and pYZ662, pYZ655 and pYZ656, pYZ663 and pYZ664, and pYZ665 and pYZ666. The final DNA constructs were then added to competent *H. influenzae* cells before these were plated onto selective plates (kanamycin 5 μg/ml for *hel*_*Hi*_ deletion and spectinomycin 20 μg/ml for *nadN*_*Hi*_ deletion). To make the double and triple mutants of *aphA*_*Hi*_, *hel*_*Hi*_*,* and *nadN*_*Hi*_*,* the chromosomal DNA of the single deletion strains of *hel*_*Hi*_ and *nadN*_*Hi*_ was used to transform the wt or *aphA*_*Hi*_ single deletion strains. The mutants were selected on sBHI agar plate supplemented with appropriate antibiotics. Besides, nicotinamide riboside was required in sBHI to support the growth of mutants with *nadN*_*Hi*_ deleted. Positive transformants were first identified *via* colony PCR and then confirmed by sequencing (using primers pYZ688 and pYZ689 for *hel*_*Hi*_ deletion and pYZ690 and pYZ691 for *nadN*_*Hi*_ deletion).

### Complementation of the triple mutant ΔaphA_Hi_Δhel_Hi_ΔnadN_Hi_ of KW20

To complement the Δ*aphA*_*Hi*_Δ*hel*_*Hi*_Δ*nadN*_*Hi*_ deletion mutant (YZ997) with *nadN*_*Hi*_, overlap PCR was utilized to construct a DNA fragment encoding sequentially the upstream 1 kbp sequence and the *nadN*_*Hi*_ gene (*via* primers pYZ661/pYZ980), the pOMP26 promotor (*via* primers pYZ655/pYZ656), an ampicillin resistance marker (*via* primers pYZ981/pYZ982 using the pACYC177 (15) plasmid as template), and finally the downstream 1 kbp sequence of *nadN*_*Hi*_ (*via* primers pYZ983/pYZ666). The final DNA construct was fed to the triple deletion mutant same as above and the complemented strain was selected on sBHI agar plate supplemented with NAD and ampicillin 1.5 μg/ml. The strain was further verified by sensitivity to spectinomycin 20 μg/ml and diagnostic PCR using primers pYZ690/pYZ691. Final Sanger sequencing did not reveal any mutation on the complemented *nadN*_*Hi*_.

### Plasmid constructions

pET28a-*aphA*_*Ec*_-his.

The primers pYZ106 and pYZ107 were used to amplify the *aphA*_*Ec*_ and constructed into the plasmid vector pET28a *via* the NcoI and NdeI restriction sites.

pET28a-his-*aphA*_*Ec*_

The primers pYZ194 and pYZ195 were used to amplify the *aphA*_*Ec*_ and constructed into the plasmid vector pET28a *via* the NcoI and NdeI restriction sites.

pET24d-his-sumo-*aphA*_*Ec*_

The primers pYZ368 and pYZ369 were used to amplify the *aphA*_*Ec*_ and constructed into the plasmid vector pET24d (Novagene) *via* the BamHI and HindIII restriction sites. Then, the linker region between *aphA* and sumo was removed *via* quickchange mutagenesis by using primers pYZ370 and pYZ371.

pET28a-his-*aphA*_*Hi*_

The primers pYZ198 and pYZ199 were used to amplify the *aphA*_*Hi*_ and constructed into the plasmid vector pET28a *via* the NcoI and NdeI restriction sites.

pET28a-his-*hel*_*Hi*_

The primers pYZ200 and pYZ201 were used to amplify the *hel*_*Hi*_ and constructed into the plasmid vector pET28a *via* the NcoI and NdeI restriction sites.

pET24d-his-sumo-*hel*_*Hi*_

The primers pYZ372 and pYZ373 were used to amplify the *hel*_*Ec*_ and constructed into the plasmid vector pET24d *via* the BamHI and EcoRI restriction sites. Then, the linker region between hel and sumo was removed *via* quick-change mutagenesis by using primers pYZ374 and pYZ375.

pET28a-his-*nadN*_*Hi*_

The primers pYZ790 and pYZ791 were used to amplify the *nadN*_*Hi*_ sequence with the signal peptide and constructed into the plasmid vector pET28a *via* the BamHI and NdeI restriction sites.

### Protein purification

*E. coli* strains were inoculated into LB medium supplemented with the appropriate antibiotic and incubated ON at 37 °C shaking 160 rpm. The next morning, the culture was diluted back into LB-medium (1:500 dilution) and grown at 37 °C, 160 rpm to OD_600_ = 0.6 to 0.8, before the culture was induced with 0.5 to 1 mM IPTG and incubated for 4 h at 37 °C, 160 rpm. Cells were harvested by centrifugation (5000 rpm, 10 min, 4 °C), resuspended in cold PBS and pelleted (4000 rpm, 10 min, 4 °C). The pellet was resuspended in cold lysis buffer (5% glycerol 50 mM Tris pH = 7.6, 150 mM NaCl, 10 mM imidazole) supplemented with β-mercaptoethanol and one tablet of cOmplete Protease Inhibitor Cocktail and was then lysed in a Branson sonicator for 8 min (2 min ON/4 min OFF) with an amplitude of 60%. The lysate was centrifugated (14,000 rpm, 40 min, 4 °C), and the supernatant was loaded onto Ni-NTA resins on a Poly-Prep column and allowed to drip through *via* gravity. The resins were then washed in cold wash buffer (5% glycerol 50 mM Tris pH = 7.6, 150 mM NaCl, 20 mM imidazole), eluted in 700 μl elution buffer (5% glycerol 50 mM Tris pH = 7.6, 150 mM NaCl, 500 mM imidazole) and further purified *via* the ÄKTA system on a Superdex 200 Increase 10/300 GL column.

### DRaCALA screening and assays

The ^32^P-labeled cAMP used for the DRaCALA were produced as described previously (18). The screening of the cAMP-binding proteins from the ASKA *E. coli* ORFeome library was performed essentially the same as in (18, 21). For determining the dissociation constant of cAMP binding to AphA, purified AphA protein was mixed with ^32^P-cAMP in a binding buffer composed of 40 mM Tris (pH 7.5), 100 mM NaCl, and 10 mM MgCl_2_. This mixture was next serial diluted in the same binding buffer containing ^32^P-cAMP (2 nM), and the samples were then incubated at room temperature for 5 min before they were spotted on a nitrocellulose membrane and visualized in a Typhoon FLA-7000 phosphorimager. For DRaCALA competition assays, the cold cAMP, AMP, or GMP at varied concentrations were individually incubated with AphA and ^32^P-cAMP (2 nM) in the same binding buffer as above. Samples were then spotted and visualized as described above. The fraction of bound was quantified as described previously (18).

### Electrophoretic mobility shift assay

A piece of *H. influenzae* chromosomal DNA (200 bp) containing the uptake sequence ACCGCACTT was PCR amplified using primers pYZ609/pYZ610, which are labeled with cy5. MAP7 DNA (45) was used as template. The DNA was subsequently purified from an agarose gel using Monarch Gel Extraction Kit. For the electrophoretic mobility shift assay, purified AphA_Hi_ protein was incubated with this DNA in a buffer composed of 20 mM Tris–HCl (pH 7.5), 100 mM KCl, 2 mM MgCl_2_, 1 mM β-mercaptoethanol, 50 μg/ml bovine serum albumin, and 0.1 mg/ml salmon sperm DNA in a total volume of 20 μl. Following 20 min of incubation at 37 °C, 3 μl 80% glycerol was mixed into the samples that were next loaded into a 5% Mini-PROTEAN gel (Bio-Rad). The gel was run at 40 V for 10 min, then at 80 V for 1 h and was finally visualized in an Image Quant LAS4000 scanner.

### Biochemical assays

To assess the enzymatic reaction of AphA, kinetic assays were performed using pPNN as the substrate as in (27). Freshly purified AphA protein was resuspended in a buffer consisting of 50 mM sodium acetate (pH 5.6) and 0.01% Triton X-100 and was subsequently transferred into a reaction buffer composed of 50 mM sodium acetate (pH 5.6), 0.1 M NaCl, 1 mM MgCl_2_, 0.01% Triton X-100, and varying concentrations of cAMP. The enzymatic reaction was started by adding varying concentrations pPNN to the reaction mixture and was terminated by transferring 60 μl of the reaction mixture into 100 μl 3 M NaOH in a clear Greiner flat bottom 96-well plate at defined timepoints. The amount of reaction product generated from pNPP was quantitated in a plate reader at 405 nm.

### Statistical analysis

GraphPad Prism v.8 (https://www.graphpad.com) was used throughout to analyze the data.

## Data availability

All data are contained within this manuscript.

## Supporting information

This article contains supporting information.

## Conflict of interest

The authors declare that they have no conflicts of interest with the contents of this article.
