# Supplementary material for: cAMP competitively inhibits periplasmic phosphatases to coordinate nutritional growth with competence of Haemophilus influenzae
Source: J Biol Chem. 2023 Oct 29;299(12):105404. doi: 10.1016/j.jbc.2023.105404 (PMC10694654; doi:10.1016/j.jbc.2023.105404)
Supplement: Table S1 [file mmc2.docx]

**Table S1: Bacterial strains used in this study**

| **Strain** | **Relevant features** | **Reference** |
| --- | --- | --- |
| YZ9 | DH5α pET28a, Kan | Novagene |
| YZ576 | pET24d; Kan | Novagene |
| YZ16 | BL21 DE3 | Laboratory stock |
| YZ142 | MG1655, wild type *E. coli* | Laboratory stock |
| YZ308 | pCA530-Ulp1-His, Kan | Laboratory stock |
| YZ272 | Rd KW20, wild type *H. influenzae* | Laboratory stock |
| YZ97 | Bl21 DE3 pET28a-*aphA*-his | This study |
| YZ253 | Bl21 DE3 pET28a-his-*aphA_Ec_*, Kan | This study |
| YZ577 | Bl21 DE3 pET28a-his-sumo-*aphA_Ec_*, Kan | This study |
| YZ254 | Bl21 DE3 pET28a-his-*aphA_Hi_*, Kan | This study |
| YZ255 | Bl21 DE3 pET28a-his-*hel_Hi_*, Kan | This study |
| YZ579 | Bl21 DE3 pET28a-his-sumo-*hel_Hi_*, Kan | This study |
| YZ1340 | Bl21 DE3 pET28a-his-*nadN_Hi_*, Kan | This study |
| YZ278 | Rd KW20 *aphA::cam*, Cam | This study |
| YZ991 | Rd KW20 ∆*hel::kan*, Kan | This study |
| YZ993 | Rd KW20 ∆*aphA::cam;* ∆*hel::kan*, Cam, Kan | This study |
| YZ995 | Rd KW20 ∆*hel::kan;* ∆*nadN::spec*, Kan, Spec | This study |
| YZ997 | Rd KW20 ∆*aphA::cam;* ∆*hel::kan;* ∆*nadN::spec*, Cam, Kan, Spec | This study |
| YZ1080 | Rd KW20, Nov | This study |
| YZ1627 | Rd KW20 ∆*aphA::cam*, ∆*hel::kan*, *nadN::amp*  Cam, Kan, Amp | This study |
