# Supplementary material for: cAMP competitively inhibits periplasmic phosphatases to coordinate nutritional growth with competence of Haemophilus influenzae
Source: J Biol Chem. 2023 Oct 29;299(12):105404. doi: 10.1016/j.jbc.2023.105404 (PMC10694654; doi:10.1016/j.jbc.2023.105404)
Supplement: Table S2 [file mmc3.docx]

**Table S2: Primers used in this study**

| **Number** | **Name** | **Sequence** |
| --- | --- | --- |
| pYZ106 | NcoI-aphA-F | CATGccATGgCCTCATCTCCTTCACCGCTT |
| pYZ107 | NdeI-aphA.his-R | GGAATTCcatatgTTAgtgatgatgatgatgatgGTATTCTGAATTGACGATCA |
| pYZ194 | NcoI-His.aphA-F | CATGccATGgcccatcatcatcatcatcaCTCATCTCCTTCACCGCTTA |
| pYZ195 | NdeI-aphA-R | GGAATTCcatatgTTAGTATTCTGAATTGACGATCA |
| pYZ198 | NcoI-His.AphAHi-F | CATGccATGgcccatcatcatcatcatcacGGCAAAACAGAACCTTATAC |
| pYZ199 | NdeI-His.AphAHi-R | GGAATTCcatatgttaATAGCTTGAATTGATTAAC |
| pYZ200 | NcoI-His.helHi-F | CATGccATGgcccatcatcatcatcatCATGCAAATATGCAATTACAAC |
| pYZ201 | NdeI-His.helHi-R | GGAATTCcatatgTTATTTACCATCCCAAGCTTG |
| pYZ368 | BamHI-aphA-F | CGggatcccgcAAGATCACACAGGC |
| pYZ369 | HindIII-aphA-R | cccAAGCTTTCAGTATTCTGAATTGACGATcac |
| pYZ370 | QCSUMOaphAfus-F | GTGGGAGAaagatcacacaggcaatcagt |
| pYZ371 | QCSUMOaphAfus-R | gtgatctttctcccaccaatctgttcg |
| pYZ372 | BamHI-hel-F | cgGGATCCCATGCAAATATGCAATTACAACAAC |
| pYZ373 | EcoRI-hel-R | gGAATTCTTATTTACCATCCCAAGCTTGTAC |
| pYZ374 | QCSUMOhelfus-F | ttggtgggCATGCAAATATGCAATTACAACA |
| pYZ375 | QCSUMOhelfus-R | TTTGCATGcccaccaatctgttcgC |
| pYZ589 | aphA-upstream-F | ATTCCACCGCACTTTTTAGC |
| pYZ590 | aphA-his-R | GTGGTGGTGGTGGTGGTGATAGCTTGAATTGATTAACACTT |
| pYZ591 | aphA-his-F | CACCACCACCACCACTAATAAATTGAACTTTTTTCTTCATC |
| pYZ592 | aphA-downstream-R | GCGGTATAGGTGTAAATAAAAATG |
| pYZ595 | aphA-his-seq-F | GTGTTACGGTGAAAACCTGG |
| pYZ596 | aphA-his-seq-R | ATCAATGCGGAAGCTATACG |
| pYZ609 | Cy5-uptake-F | CGCCAATCTTTCGCTAATTC |
| pYZ610 | Cy5-uptake-R | CGTAATGAAAGGTAATGCTCG |
| pYZ653 | Δhel-F1 | GGAAAGATTTAGACGAGCTTC |
| pYZ654 | Δhel-R1 | AAAAAGTTCAATTTAAAGTGCGGTCATTTTTAAC |
| pYZ655 | Δhel/ΔnadN-F2 | TAAATTGAACTTTTTTCTTCATC |
| pYZ656 | Δhel/ΔnadN-R2 | TTGATAAATATCCTTAATTAAATGATG |
| pYZ657 | Δhel-F3 | AAGGATATTTATCAAATGAGCCATATTCAACGG |
| pYZ658 | Δhel-R3 | TGAAAGTTTTACCAATTAGAAAAACTCATCGAGCA |
| pYZ659 | Δhel-F4 | TTGGTAAAACTTTCATCATGTTAC |
| pYZ660 | Δhel-R4 | ATAATTTGACGGTCTGAAATAAC |
| pYZ661 | ΔnadN-F1 | ATAGACAAAAAGCCTGCG |
| pYZ662 | ΔnadN-R1 | AAAAAGTTCAATTTAAAAAACTCCTATTGGTAAGGG |
| pYZ663 | ΔnadN-F3 | AAGGATATTTATCAAATGAGGGAAGCGGTGAT |
| pYZ664 | ΔnadN-R3 | ATGCATCAGTTGAAGTTATTTGCCGACTACCTTG |
| pYZ665 | ΔnadN-F4 | CTTCAACTGATGCATTACC |
| pYZ666 | ΔnadN-R4 | GGATTCGTTACAATCATCAC |
| pYZ688 | Δhel-conf-F | GGCAACAGGTGTATTTACTG |
| pYZ689 | Δhel-conf-R | AAAACTGAGCTTAGCCCCT |
| pYZ690 | ΔnadN-conf-F | ACAAAACCTTTCAAGCAATG |
| pYZ691 | ΔnadN-conf-R | GATTATCGCAATAAATCACC |
| pYZ739 | Xhol-Cam-F | CCGCTCGAGGCGCGCCTACCTGT |
| pYZ740 | XbaI-Cam-R | GCTCTAGACCCTGCCACTCATCGC |
| pYZ790 | NdeI-nadN-F | GGAATTCcatatgGCACTCAGTGCATTTGC |
| pYZ791 | BamHI-nadN-R | CGggatccttaTTTTTTAGGTAATGCATCAG |
| pYZ980 | nadN-upstream-R | AAAAAGTTCAATTTAttaTTTTTTAGGTAATGCATCAG |
| pYZ981 | Amp-F | AAGGATATTTATCAAatgagtattcaacatttccg |
| pYZ982 | Amp-R | TTACCAATGCTTAATCAGTGAG |
| pYZ983 | nadN-downstream-F | attaagcattggtaaAATTGAAAAATAGGCTAAAAATAG |
